# Direct acceleration of electrons by a CO_2_ laser in a curved plasma waveguide

**DOI:** 10.1038/srep28147

**Published:** 2016-06-20

**Authors:** Longqing Yi, Alexander Pukhov, Baifei Shen

**Affiliations:** 1 Institut fuer Theoretische Physik I, Heinrich-Heine-Universitaet Duesseldorf, Duesseldorf, 40225 Germany; 2 State Key Laboratory of High Field Laser Physics, Shanghai Institute of Optics and Fine Mechanics, Chinese Academy of Sciences, P.O. Box 800-211, Shanghai 201800, China; 3 Collaborative Innovation Center of IFSA (CICIFSA), Shanghai Jiao Tong University, Shanghai 200240, China

## Abstract

Laser plasma interaction with micro-engineered targets at relativistic intensities has been greatly promoted by recent progress in the high contrast lasers and the manufacture of advanced micro- and nano-structures. This opens new possibilities for the physics of laser-matter interaction. Here we propose a novel approach that leverages the advantages of high-pressure CO_2_ laser, laser-waveguide interaction, as well as micro-engineered plasma structure to accelerate electrons to peak energy greater than 1 GeV with narrow slice energy spread (~1%) and high overall efficiency. The acceleration gradient is 26 GV/m for a 1.3 TW CO_2_ laser system. The micro-bunching of a long electron beam leads to the generation of a chain of ultrashort electron bunches with the duration roughly equal to half-laser-cycle. These results open a way for developing a compact and economic electron source for diverse applications.

Laser wakefield acceleration^1^ of particles to relativistic energies has been greatly promoted by the invention of chirped-pulse-amplification (CPA)^2^ more than twenty years ago. The ultrashort laser pulses with huge peak powers enabled by the CPA technique allowed for quasimonoenergetic electron bunches to be generated in underdense plasmas in so-called bubble regime^3^,^4^,^5^,^6^,^7^,^8^ of laser wakefield. Pioneering experiments had reported that electrons with a few percent energy spread and sub-milliradian divergences beyond 4.2 GeV can be produced^9^, which demonstrates the impressive progress in plasma-based acceleration. However, a significant drawback is the traditional CPA lasers use TiSa crystals which deliver an average power of a few Watts only at a low overall efficiency^10^. On the other hand, well-known for its industrial applications, the overall efficiency (5–20% from wall plug) of CO_2_ laser is among the highest of all lasers. Hence, it is the most economic choice when considering high energy physics applications, where high luminosities are usually required.

Nowadays, high-pressure CO_2_ laser has already reached multi-Terawatt-level^11^ and been successfully applied for a series of proton acceleration experiments^12^,^13^. However, it has been less progresses in CO_2_ laser-driven wakefield acceleration^14^,^15^, mainly because of the difficulty with building ultra-short CO_2_ laser system. In general, it is well known that the longitudinal dimension of the driver of plasma wakefield should be comparable to plasma wavelength in order to resonantly excite a bubble^3^,^8^,^16^, such that for the typical CO_2_ laser pulse duration (*τ* ~ 10 ps), an extremely low-density plasma (*n*_*e*_ ~ 10^13^ cm^−3^) is required, which is of little interest to the accelerator community since the maximum acceleration gradient (i.e. wave breaking field) is on the same order of magnitude with the conventional RF accelerators.

In parallel, direct laser acceleration (DLA) offers an attractive alternative^17^,^18^, where no threshold intensity^19^ and no limitiations on the pulse duration. Normally, a waveguide can be used to guide laser pulses over distances much larger than the Rayleigh length 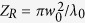 (*w*_0_ is the spot size and *λ*_0_ is the laser wavelength), while simultaneously, transverse magnetic (TM) optical modes are excited in the channel. The co-propagating electrons in a proper phase can be accelerated with a peak longitudinal electric field that can be estimated by^20^,^21^,^22^


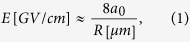


where *a*_0_ = *eE*_0_/*m*_*e*_*cω*_0_ is the normalized laser amplitude, *c* is the light velocity in vacuum *m*_*e*_ is the mass of an electron, *e* is the unit charge, *ω*_0_ is the frequency of laser, and *R* is the radius of waveguide in *μm*. For a 1.3 TW CO_2_ laser pulse with *a*_0_ = 5 and channel radius *R* = 6*λ*_0_ ≈ 64 *μm*, the peak acceleration gradient is roughly 0.64 GV/cm, which compare favorably to laser wakefield acceleration with ultra-short laser pulses at the same power level.

However, the slippage between laser phase velocity and the electron velocity (essentially *c*) forbids the electrons to stay in the acceleration phase, which sets a limit on the maximum energy that can be aquired. Historically, periodical grating surfaces^23^,^24^ and neutral gas filling^25^ have been proposed to solve the dephasing problem, but none of them can survive the relativistic intensities required for high acceleration gradient even for the pulse duration. In fully ionized plasma channel, the phase velocity of laser is superluminal. A corrugated plasma waveguide has been proposed as ultrahigh intensity optical slow-wave structure, where net energy gain can be achieved using a radially polarized laser propagating in a density-modulated gas jet^19^. More recently, owing to the advancements in laser pulse cleaning techniques^26^,^27^ and 3D direct laser writing (DLW) of materials^28^, laser interaction with fine plasma structure is drawing more and more attention. The micro- and nano-structured plasma targets have been introduced to manipulate laser matter interactions^22^,^29^,^30^,^31^. Simulations suggest that the longitudinal electric field in excess of 1 TV/m can be achieved in an overdense micro-plasma-waveguide^22^. An taylored plasma microstructure that can overcome the phase slippage would allow for enormous acceleration gradients.

## Results

### Laser propagation and electron motion in a Curved plasma waveguide (CPW)

**Figure 1:**
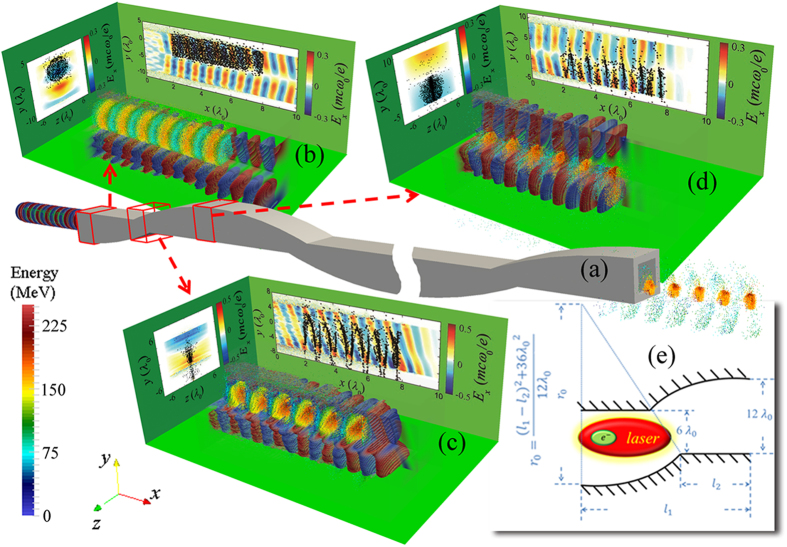
Sketch of direct laser acceleration of electron in a CPW. (**a**) A linearly polarized CO_2_ laser and a relativistic electron beam are injected into a CPW from the left side. The electrons located in the right phase can be accelerated by the TM modes, resulting in the generation of a chain of energetic ultrashort electron bunches (right exit of CPW). (**b**–**d**) Shows the longitudinal electric field and electron motion at different propagating distances (marked by the red cube-frame in (**a**)), where a quarter of *E*_*x*_ field are removed to avoid overlapping, and the electron energy is presented by the color. The figure on the back and left walls in (**b**–**d**) presents the *E*_*x*_ field and electron position (black dots) at longitudinal slice *z* = 0 and transverse cross-section *x* = 6.5*λ*_0_, respectively. The waveguide is properly designed with a curvature in the polarization direction, detail dimension of half of the CPW period is shown in (**e**) for x-y plain (the waveguide is uniform along z-direction, and the size in z dimension is 12*λ*_0_ in the presented 3D PIC simulation). The *l*_1_ is the half-CPW curvature period, and the ratio of *l*_1_ and *l*_2_ is optimized according to the simulation (*l*_2_ = 0.442*l*_1_). If *l*_1_ matches the dephasing length, the electron bunch can continuously gain energy until it overtakes the entire laser pulse.

In this letter, we propose a novel electron acceleration scheme using DLA in a curved plasma channel that is capable of generating energetic (>1 GeV) ultra-short (duration ~ half-laser-cycle) electron bunch chain with slice energy spread ~1%. These high-quality electron beams can be widely applied in high energy physics, study of atomic and molecular dynamics and generating coherent x-rays. In the presented study, A CPW is used to overcome the phase slippage as shown in Fig. 1. Inspired by the fact that the longitudinal electric field in the CPW is anti-symmetric with respect to the propagation axis in the polarization direction (as shown in Fig. 1(b–d)), we properly design the spatial period of CPW to match the dephasing length of electrons. So that the relative displacement between electrons and laser field in the longitudinal and transverse directions keeps the electrons in the accelerating phase (for most of the time), enabling continuous energy gain of witness beam until it overtakes the entire laser pulse. In addition, a linearly polarized CO_2_ laser beam is employed as the driver, not only due to the high overall efficiency discussed above, but also because a long infrared wavelength enables a large acceleration bracket, which increases the number of particles per bunch. Nevertheless, the wavelength of drive laser is not mandatory, it should be carefully chosen according to the applications.

### Electron acceleration process and the energy gain

Here we first demonstrate the acceleration process with three dimensional (3D) particle-in-cell (PIC) simulations using the code VLPL^32^, the parameters can be found in **Methods**. The CPW is constituted with an overdense slab and an arc, the detailed dimensions for the structure are shown in Fig. 1(e). Due to the up-and-down motion of laser pulse in CPW tends to push the electrons out of the waveguide, especially in the first CPW period (since the gamma factor of electrons is smaller in the beginning), the initial energy of the electron bunch is chosen as 100 MeV to ensure that an essential part of the injected electrons could be stably accelerated by The TM modes. Figure 1(b–d) show the relative motion of the electron bunch and longitudinal electric field in half of the CPW period, which indicate the transverse motion of the guided laser beam perfectly compensates the phase slippage effect. As soon as the longitudinal phase slippage reaches half of laser cycle, electrons exactly fall into the acceleration phase on the opposite side of channel as expected. Moreover, since only the electrons with proper phase (*E*_*x*_ < 0) can be captured and accelerated within a long electron bunch, a chain of ultrashort electron bunches is generated. The duration of a single electron bunch is governed by the laser wavelength, an attosecond electron train can be generated if a short-wavelength laser is employed as the driver, which may be applied in the ultrafast electron diffraction and 4D microscopy^33^,^34^,^35^.

**Figure 2:**
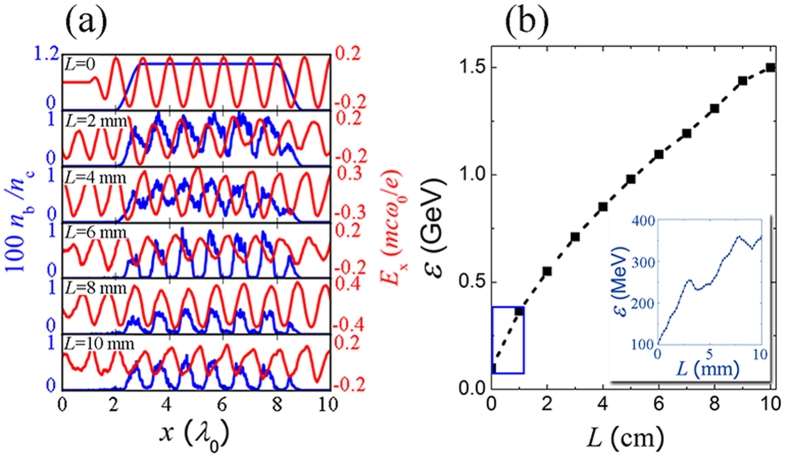
Simulation results of the electron acceleration. (**a**) The on-axis longitudinal electric field and witness electron density profile in the first CPW period at propagation distance *L* = 0, 2, 4, 6, 8, and 10 mm (**b**) peak energy of electron bunch at the end of each CPW period, the inset shows detail energy evolution in the first CPW period (blue rectangular in (**b**)).

In order to obtain a deeper insight in the laser pulse propagation and electron acceleration, we perform 2D PIC simulation on the electron acceleration over 10 centimeters (10 CPW periods). A long laser pulse (duration ~1 ps) is employed to enable the acceleration over a long distance. The on-axis longitudinal electric field and witness electron density profile are plotted in Fig. 2(a) for one CPW period. One can see that the micro-bunching occurs simultaneously with the acceleration during the first 2-mm propagation in the CPW. The generated micro bunches stay in the acceleration phase for most of time.

Figure 2(b) presents the peak energy of electron bunch at the end of each CPW period, where the final electron energy attained is 1.5 GeV. The acceleration gradient decreases as the laser energy depletes in the CPW. After 10 cm propagation, the laser loses 80% of its energy. For electrons with divergence smaller than 1 mrad, the overall laser-to-electron energy efficiency is roughly 11%. The inset of Fig. 2(b) shows the peak electron energy evolution in the first CPW period. The slight energy decrease observed at *L* ≈ 3 mm and 8 mm is due to the brief passage of the witness bunch through the decelerating phase as the laser pulse is guided across the beam axis (see Figs 1(c) and 2(a)). The net energy gain in the first CPW period (1 cm) is 260 MeV that results in an average acceleration gradient of 26 GV/m, or roughly 40% of the peak electric field predicted in Eq. (1).

### Theoretical analysis of the optical modes in plasma waveguide

In the following, we try to give an estimation on the electromagnetic field and acquire basic results for dephasing distance by investigating laser propagation in a plane plasma waveguide. Further PIC simulation should be relied on to obtain optimum parameters for a real CPW. Considering a *y*-polarized laser pulse entering the plasma waveguide along *x*-axis, the waveguide has a rectangular cross-section in *y*-*z* plane (*y* = −*y*_0_ ~ *y*_0_, and *z* = −*z*_0_ ~ *z*_0_). Following the methods in refs 21,22, one can easily write the electric and magnetic fields in terms of two Hertz potentials Π^*e*^ and Π^*h*^ in Cartesian coordinate system:


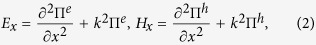



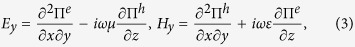



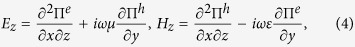


where


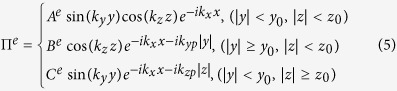



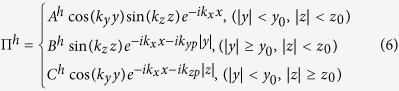


here *A*^*e*^, *B*^*e*^… etc are coefficients determined by the incident laser amplitude, 
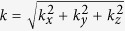
 is the total wave number in the vacuum core, and *k*_*x*_, *k*_*y*_, *k*_*z*_ are the wave numbers in each direction, *k*_*yp*_, *k*_*zp*_ are the transverse wave numbers inside the plasma channel walls. Apparently, since laser is properly guided in the *x* direction, *k*_*yp*_, *k*_*zp*_ are both imaginary.

At boundaries *y* = ±*y*_0_ and *z* = ±*z*_0_, the tangential components of both *E* and *H* should be continuous, which leads to the following eigenvalue equations,


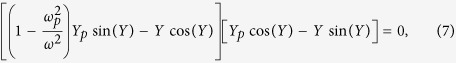



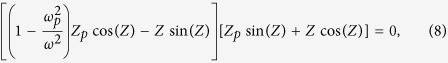


where *ω*_*p*_ is the plasma frequency, and *Y* = *k*_*y*_*y*_0_, *Y*_*p*_ = −*ik*_*yp*_*y*_0_, *Z* = *k*_*z*_*z*_0_, *Z*_*p*_ = −*ik*_*zp*_*z*_0_, which satisfy 
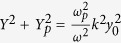
, and 
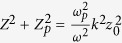
.

In 2D limit, i.e. 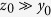, the wave number *k*_*z*_ is negligible. Let *α* be the 1st root for *Y* in Eq. (7) (*α* = 1.54), which corresponding to the lowest TM mode in the waveguide. One can write the longitudinal electric field according to Eq. (2) as


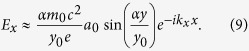


## Discussion

### A discussion on the energy spread

**Figure 3:**
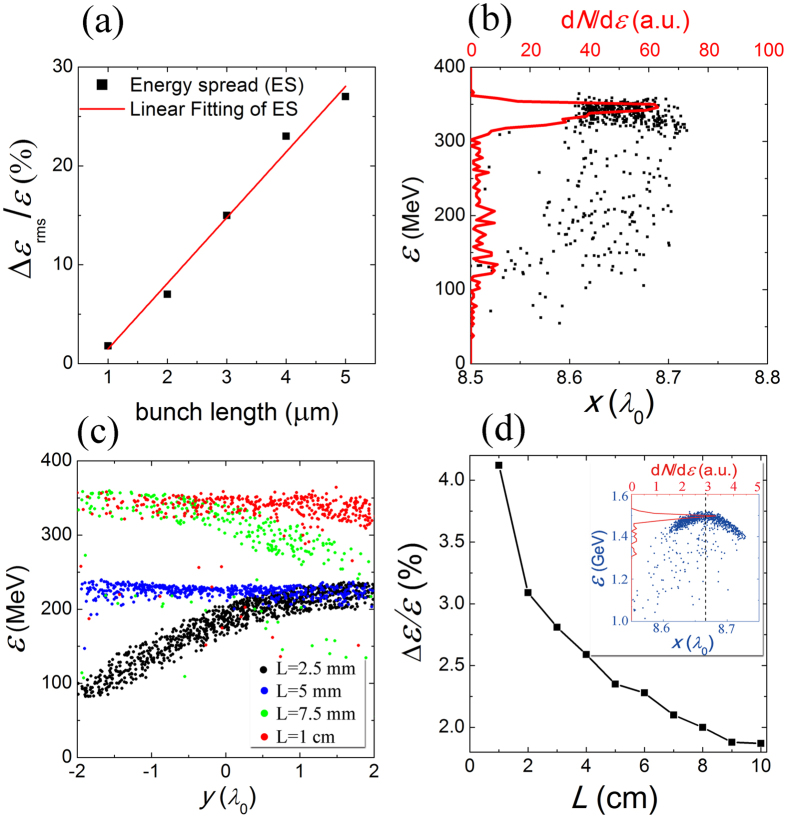
Energy spread of the accelerated electron bunch in CPW. (**a**) The r.m.s. energy spread obtained for different injected bunch durations. (**b**) Longitudinal and (**c**) transverse phase space of electrons in the first CPW period for 1-*μ*m witness duration. (**d**) The relative energy spread evolution during the 10-cm acceleration of 1-*μ*m duration witness bunch. The red line in (**b**) shows the electron energy spectrum, the different colors in (**c**) corresponding to the phase space at corresponding distance, and the inset of (**d**) presents the phase space of electrons at highest energies.

Apparently, the acceleration gradient is only uniform along the *z* –direction, the nonuniformity in x- and y-direction tends to broaden energy spread of witness beam during the acceleration. The energy spread of witness bunch can be controlled by the duration of injected electron bunch as shown in Fig. 3(a), the bunch duration above 5 *μ*m is not considered because it’s beyond the longitudinal size of each electron bunch after the micro-bunching of a long electron beam. The linear growth of energy spread is resulted from the longitudinal energy chirp owing to sinusoid distribution of acceleration gradient in Eq. (9). However, we emphasis that the slice energy spread is small (~1%) regardless the witness beam length, which suggest a high quality of the acceleration. When a short witness electron bunch (~1 *μ*m, occupies about 10% of the laser cycle) is injected into the CPW, the witness bunch gains little longitudinal energy chirp as shown in Fig. 3(b). Also, the transverse phase space map (Fig. 3(c)) illustrates that although the accelerating field varies along the y-axis, the integration in one period of CPW is uniform. So the electrons at different transverse position gains the same amount of energy in one CPW period. As a result, when a short (1 *μ*m) electron bunch is injected into the proper accelerating phase, a quasi-monoenergetic electron beam can be obtained as shown in Fig. 3(d). The relative r.m.s energy spread at highest energy (1.5 GeV) is about 2% for the whole beam, this result can be further optimized by employing shorter witness bunch. The absolute energy spread Δ*ε* increases slightly owing to longitudinal energy chirp as shown by the inset phase space map, and the r.m.s slice energy spread is 0.83%.

### A discussion on the matching condition

The above results also allow us to derive the matching condition which is crucial to the proposed scheme. In a sufficiently short propagation distance *dx*, the change in transverse size of CPW is negligible, the CPW can be treated as a plane waveguide, and the phase slippage between relativistic electrons (*v* ≈ *c*) and TM_10_ mode is 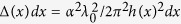, where *h*(*x*) is the CPW dimension along *y*-direction. The matching condition states that the phase slippage in half CPW period must be equal to *λ*_0_/2, i.e.


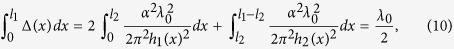


where 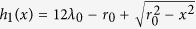 and 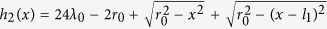 are the expressions for *h*(*x*) in the flat and curve areas, respectively.

**Figure 4:**
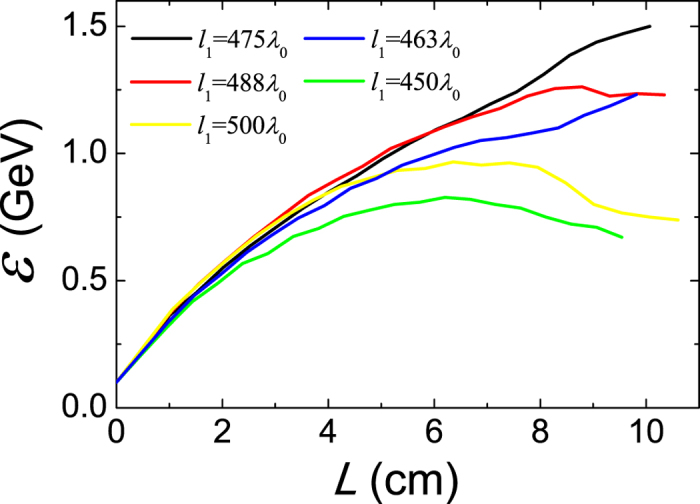
Energy gain of injected electron bunch in CPW with different period.

Equation (10) indicates the dephasing length is very sensitive to the transverse size of CPW, and it should be noted we have ignored the small transverse motion of laser pulse for simplicity. According to Eq. (10), we know that the longitudinal dimension of CPW should be chosen around the match condition *l*_1_ = *L*_*d*_ ≈ 488*λ*_0_, which roughly agrees with our numerical observations. By scanning over a range of CPW periods, we found the optimal acceleration is obtained for a slightly-shorter CPW with *l*_1_ = 475*λ*_0_ as shown in Fig. 4. Apparently the violation of matching condition lead to early saturation which limits the energy gain. The maximum energy decreases 50% for 25 *λ*_0_ deviation from the matched cases.

In conclusion, a novel DLA scheme based on CPW at high laser intensities is proposed and tested by multi-dimension PIC simulations. Our results indicate that a CPW can be used as an electron accelerator when coupled with state-of-art CO_2_ laser beams. The proposed scheme demonstrates high acceleration gradient and beam quality, which makes it a promising candidate for future tabletop accelerator design. In addition, the overall efficiency of the scheme is high because the CO_2_ laser pulses have high wall-plug efficiencies. The underlying physics is discussed using PIC simulations and theoretical analysis, the matching condition is presented, which agrees with our numerical observation. The integration of longitudinal electric field in one CPW period results in uniform accelerating structure in transverse direction. A quasi-monoenergetic electron bunch with mean energy 1.5 GeV, r.m.s. energy spread 2% can be obtained within 10-cm acceleration. Meanwhile, when a long electron beam is injected into the CPW, micro-bunching effect comes into play, which is capable of generating a chain of ultra-fast electron bunchs with the dimension of half-laser-cycle.

## Methods

### Numerical modeling of the direct laser acceleration in a CPW

Due to the computational difficulty with simulating the realistic long CO_2_ laser pulse, we perform three dimensional (3D) simulation with an relatively small window 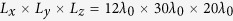 focused on the laser-electron interacting position over half of the CPW period (*L*_*acc*_ = 475*λ*_0_ ≈ 0.5 cm) to examine the electron motion between two accelerating phases. The simulation resolution is dx = 0.04*λ*_0_, dy = dz = 0.08*λ*_0_ in each direction, where *λ*_0_ = 10.6 *μm* is the wavelength of CO_2_ laser. In 2D simulations, a bigger simulation window (*L*_*x*_ × *L*_*y*_ = 32*λ*_0_ × 26*λ*_0_) and a finer resolution (dx = 0.02*λ*_0_, dy = 0.05*λ*_0_) are employed, while other parameters remain the same. A Moving window is used for both 2D and 3D simulations for computational efficiency. The particle per cell used in the simulation is 10 for 2D and 5 for 3D simulations. The absorbtion boundary for particles and periodic boundary for electric/magnetic fields are employed. The laser pulse in the window is assumed to have a trapezoidal profile in time with normalized amplitude *a*_0_ = 5, which propagates in the positive *x* direction. The plasma channel wall has a uniform density of *n* = 3*n*_*c*_, where 
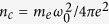
 is the critical density. It should be noted although the density of MPW is limited by computational efficiency, in real experiments, the laser can hardly penetrate into the area with *n* > 3*n*_*c*_ due to finite density gradients. In addition, the CPW contains a uniform low-density (10^15^ cm^−3^) plasma to provide necessary focusing for witness particles, it has little influence to the accelerating field and laser propagation. The witness electron bunch has a flat density profile in *x* direction (duration ≈ 4*λ*_0_) and a Gaussian profile (FWHM ≈ 2*λ*_0_) in transverse direction.

## Additional Information

**How to cite this article**: Yi, L. *et al*. Direct acceleration of electrons by a CO_2_ laser in a curved plasma waveguide. *Sci. Rep.* **6**, 28147; doi: 10.1038/srep28147 (2016).
